# Using demographic data to better interpret pitfall trap catches

**DOI:** 10.3897/zookeys.100.1530

**Published:** 2011-05-20

**Authors:** Andrey V. Matalin, Kirill V. Makarov

**Affiliations:** Zoology & Ecology Department, Moscow State Pedagogical University, Moscow, Russia

**Keywords:** Carabidae, abundance, community, resident, migratory, sporadic, residential habitats, transit habitats, labile component, stable component, zonal sites, azonal sites

## Abstract

The results of pitfall trapping are often interpreted as abundance in a particular habitat. At the same time, there are numerous cases of almost unrealistically high catches of ground beetles in seemingly unsuitable sites. The correlation of catches by pitfall trapping with the true distribution and abundance of Carabidae needs corroboration. During a full year survey in 2006/07 in the Lake Elton region (Volgograd Area, Russia), 175 species of ground beetles were trapped. Considering the differences in demographic structure of the local populations, and not their abundances, three groups of species were recognized: residents, migrants and sporadic. In residents, the demographic structure of local populations is complete, and their habitats can be considered “residential”. In migrants and sporadic species, the demographic structure of the local populations is incomplete, and their habitats can be considered “transit”. Residents interact both with their prey and with each other in a particular habitat. Sporadic species are hardly important to a carabid community because of their low abundances. The contribution of migrants to the structure of carabid communities is not apparent and requires additional research. Migrants and sporadic species represent a “labile” component in ground beetles communities, as opposed to a “stable” component, represented by residents. The variability of the labile component substantially limits our interpretation of species diversity in carabid communities. Thus, the criteria for determining the most abundant, or dominant species inevitably vary because the abundance of migrants in some cases can be one order of magnitude higher than that of residents. The results of pitfall trapping adequately reflect the state of carabid communities only in zonal habitats, while azonal and disturbed habitats are merely transit ones for many species of ground beetles. A study of the demographic structure of local populations and assessment of the migratory/residential status of particular carabid species are potential ways of increasing the reliability of pitfall trap information.

## Introduction

Pitfall trapping is one of the most commonly used techniques to quantify terrestrial arthropods (Barber 1931). The simplicity of the method and the possibility of data standardization are the main advantages of their application in numerous entomological studies. Pitfall trapping is easy, and as such arthropods can be captured in different places at the same time. This explains the extensive use of pitfall traps in ecological investigations of ground beetles (Scherney 1959; Skuhravý 1959; Novák 1964; Kabacik-Wasylik 1970; Tietze 1973; den Boer 1977; Brandmayr and Zetto Brandmayr 1986; Østbye and Hägvar 1996; Gryuntal 2008; Makarov and Matalin 2009).

However, doubts concerning the reliability of the obtained results were already expressed during the first pitfall trap studies and have been discussed subsequently (for example, see Adis 1979). Numerous factors have been found to affect pitfall trap catches, such as, the size of a trap and its inlet (Luff 1975; Waage 1985; Work et al. 2002; Koivula et al. 2003), the colour of a trap (Buchholz et al. 2010), the presence and type of preservative (Luff 1968; Feoktistov 1980; Gryuntal 1982; Karpova and Matalin 1992; Weeks and McIntyre 1997) and the ways of setting traps across habitats (Greenslade 1964; Perner and Schueler 2004; Korczycski and Sienkiewicz 2006). In addition, the mobility of beetles in relation to both their physiological condition and the environment vary widely in the course of a season and between seasons (den Boer 1977; van Huizen 1977, 1979; Baars 1979; Matalin 1994, 1997, 2003; Desender 2000).

Towards the second half of the 20th century it became clear that pitfall trapping reflected not as much the abundance as the locomotor activity of beetles. Numerous steps have been taken to increase the reliability of the results of catches: changes in trap construction (Reeves 1980; Boucher 1981; Kuschka et al. 1987; Loreau 1987; Dufrêne 1988; Makarov and Tshernyakhovskaya 1990; Karpova and Matalin 1992; Kuschka 1998) and in the type of preservative used (Louda 1970; Feoktistov 1980; Gryuntal 1982; Pekar 2002), exhaustive catches from enclosed areas (Kudrin 1971; Gryuntal 1981; Desender and Maelfait 1986), the calculation of correction coefficients from the re-trapping of marked specimens (Holland and Smith 1991; Raworth and Choi 2001), and the comparisons of dynamic (pitfall trapping) and static (standard soil fauna quadrate sampling) population densities (Kudrin 1966; Arnoldi et al. 1972; Desender and Segers 1985; Spence and Niemelä 1994). In spite of these important advances, standard pitfall trapping has ‘de facto’ become a standard technique used in synecological investigations of Carabidae.

At the same time, when pitfall-trapped data are interpreted, the beetles’ migratory capacities are often ignored. This is because there is no universal technique for quantitatively estimating beetle locomotion (den Boer 1977; Prisnyi 1987). Interpretation of life cycles to evaluate the demographic structure of local populations can provide a new approach to solving this problem. For example, a significantly deficient demographic structure recently observed in some carabid species in agricultural or disturbed habitats shows that in many places the populations are represented only by certain ‘age groups’ (Borkowski and Szyszko 1984; Wallin 1989; Makarov and Tshernyakhovskaya 1989; Tshernyakhovskaya 1990; Khotuleva 1997). According to data obtained by Bokhovko (2006), five of the 11 dominant carabid species from arable soils in the Kuban Region, southern Russia, demonstrated high abundance levels, coupled with incomplete demographic spectra. For example, in semi-centennial forest belts as well as in alfalfa fields, about 80% of the dominants completed their development. On the other hand, in corn fields and in a forest belt with *Robinia*, about 75% of the carabid beetles did not complete their full life cycle.

The last case clearly illustrates the probable scales of migration in Carabidae, showing that populations are often incapable of reproducing in such environments. However, it still remains unclear whether this situation is general or not. We can assume that the proportion of species with incomplete demographic spectra represented in pitfall traps is higher in disturbed habitats, while in undisturbed or moderately disturbed habitats, the sex and age structures of the populations are more or less balanced.

In the present study, we highlight a key methodological problem that the actual community structure (e.g., the roles of individual species) cannot be understood based on pitfall counts alone. We also demonstrate how demographic analysis can be used to address this problem.

## Material and methods

Ground beetle communities in the Lake Elton region, Volgograd Area, south-eastern Russia (49o12.47’N, 46o39.75’E) were studied in 2006–2007. Lake Elton is situated within the Botkul-Bulukhta drainless desert depression, which belongs to the Caspian Lowland. A strongly pronounced salt-dome structure is characteristic of this region, and desert steppes are typical plant associations in most of the habitats present (Nekrutkina 2006; Safronova 2006). Dense reedbeds occur in the river valleys, in gullies at lakesides there are trees and shrubs, while lakesides near the mouth of most large rivers are characterised by salt-marshes. Near the village of Elton, all desert steppes are fragmented or transformed into pastures.

Pitfall trapping was conducted in 10 habitats: six zonal characteristic of this particular biogeographical area, and four azonal present in a variety of biogeographical areas (Walter 1973; Chernov 1975). Three selected habitats were located near the village of Elton, while seven were placed on the north-western shore of Lake Elton, on the right bank of the River Khara (for more details see Makarov and Matalin 2009). Zonal habitats were represented by sagebrush and sagebrush-grassland steppe with varying degrees of anthropogenic disturbances (strong near Elton village, moderate on the northern slope of Mt. Ulagan, and weak in the watershed of River Khara). Azonal habitats were chosen along salinity and solar irradiation gradients (strong in the lakeside salt-marsh, moderate in the salina on the floodplain terrace of River Khara, and weak in reedbeds along River Khara).

Plastic cups of 0.5 L capacity and 95 mm upper diameter containing 4% formaldehyde solution as a preservative were used. In each habitat, 10 traps were arranged along transects at 10 m intervals. The traps were checked every ten days from 10 May to 31 October in 2006 and from 1 April to 10 May in 2007.

All captured carabids were dissected. Based on gonad condition (Gilbert 1956, Skuhravý 1959, van Heerdt et al. 1976, Wallin 1989), as well as on the degree of wear-and-tear of the mandibles, claws and cuticle (Houston 1981, Brandmayr and Zetto Brandmayr 1986, Butterfield 1986, Davies 1987), six physiological states in the adults of both sexes were distinguished.

### Teneral

Recently emerged beetles with soft and pale cuticle; mandibles and claws sharp. Ovaries thin, white or translucent without any trace of developing oocytes; corpora lutea absent; lateral oviducts very thin. Testes thin and dull or relatively large and white; accessory glands always thin and poorly visible.

### Immature.

Cuticle fully hardened and coloured; mandibles and claws pointed. Ovaries compact, opaque and white, with or without distinctly visible oocytes, but always without ripe eggs; corpora lutea absent; lateral oviducts long and thin. Testes opaque and white; accessory glands no longer than half of the abdominal length, occupying less than a third of the abdominal space.

### Mature of parental generation.

Cuticle slightly worn; mandibles and claws hardly or distinctly dulled. Ovaries with ripe eggs; corpora lutea absent or yellowish, hardly visible; lateral oviducts wide. Testes large and white or cream-coloured; accessory glands long and white or light-yellow, filling more than three-quarters of the abdominal space.

### Mature of ancestral generations.

Cuticle clearly worn; mandibles and claws dull. Ovaries with ripe eggs; corpora lutea distinctly light or dark brown; lateral oviducts wide. Testes large and cream-coloured; accessory glands long and cream-coloured or light-brown, filling more than three-quarters of the abdominal space.

### Spent of parental generation.

Cuticle clearly worn; mandibles and claws as a rule distinctly dull. Ovaries compactly opaque and cream-coloured, without ripe eggs; corpora lutea clearly visible and dark brown, often deposited above last developing oocytes; lateral oviducts wide. Testes medium-sized or relatively small (regressed), opaque and cream-coloured or yellow; accessory glands thin opaque and yellow or light-brown, occupying less than a third of the abdominal space.

### Spent of ancestral generations.

Cuticle very worn; mandibles and claws blunt. Ovaries compactly opaque and cream-coloured or light-brown, without ripe eggs; corpora lutea clearly visible and dark brown, as a rule deposited under the developing oocytes; lateral oviducts wide. Testes medium-sized or relatively small (regressed), opaque and yellow or brown; accessory glands thin opaque and yellow, yellow-orange or brown, occupying less than a third of the abdominal space.

The separation between parental and ancestral generations was somewhat subjective and should be interpreted with caution. However, in most cases this separation was not required for the reasonable interpretation of demographic structures of the studied populations.

## Results

Detection of the chronology of the maximum activity of the above-mentioned groups of specimens in the key stages of their life cycles as a result of feeding, reproduction or preparation for hibernation, forms the basis of our analysis. In such an approach, the quantitative recording of eggs, larvae, and pupae is not required. Moreover, we can evaluate the demographic spectra of a local population from small numbers (several dozen) of individuals.

**Figure 1.**
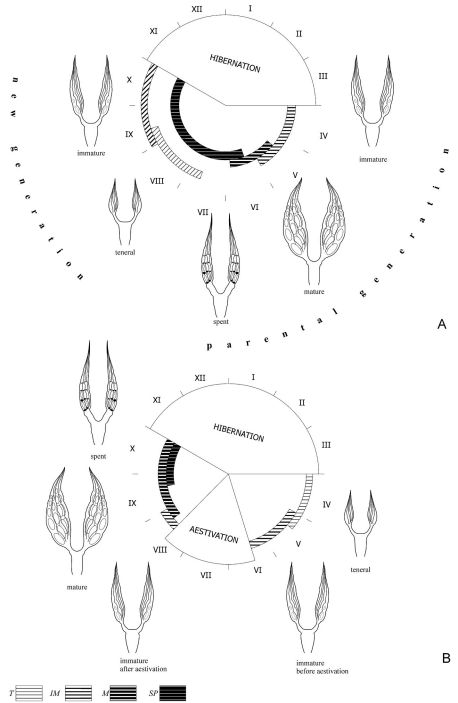
Chronology of changes in periods of activity of individual ‘age’ groups, characterised by female gonad condition, in ‘spring’ (**A**) and ‘autumn’ (**B**) breeding carabid beetles (T – teneral, Im – immature, M – mature, Sp – spent beetles).

**Figure 2.**
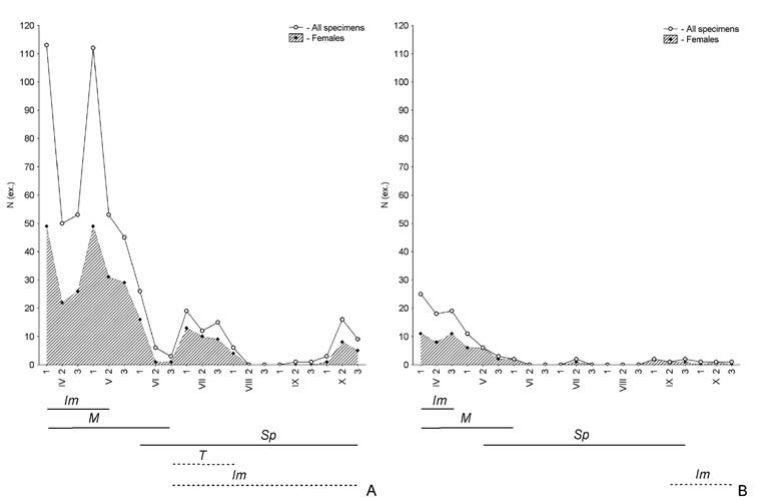
Seasonal dynamics of activity, as well as the age structure of the populations of *Pogonus transfuga* (**A**) and *Brachinus hamatus* (**B**) from reedbeds along the River Khara, combined data for 2006/07 (T – teneral, Im – immature, M – mature, Sp – spent beetles; solid lines below graphs parental generation, dashed lines below graphs – new generation; **N** (ex.) – number of specimens; **1**, **2**, **3** – first, second and third ten-day periods per month, respectively).

In ‘spring breeders’ (Types 1 and 2 according to Thiele 1977), such a chronological series represents: immature of parental generation after hibernation → mature of parental generation → spent of parental generation → teneral of new generation → immature of new generation prior to hibernation (Fig. 1A). During this sequence, the abundance of species can be high or low. For example, in the reedbeds along the River Khara in early spring, peaks of abundance in the populations of *Pogonus transfuga* and *Brachinus hamatus* were observed. However, in the former species abundance reached 112–113 individuals in early April and early May (Fig. 2A), while in the latter species, abundance during April was less than 25 individuals (Fig. 2B). In spite of this, both species are characterised by a complete demographic spectrum.

**Figure 3.**
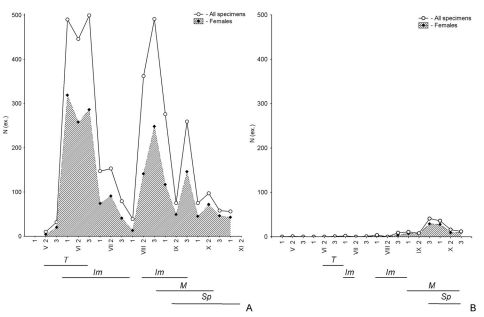
Seasonal dynamics of activity, as well as the age structure of the populations of *Calathus ambiguus* from grass-forb steppe with *Amygdalus nana* (**A**) and *Pseudotaphoxenus rufitarsis major* from sagebrush-grassland desert steppe on the northern slope of Ulagan Mountain (**B**), in 2006 (breaks in the periods of activity of immature specimens correspond to the time of aestivation parapause; see Figure 2 for further explanations).

In ‘autumn breeders’ (Type 4 according to Thiele 1977), the chronological series is as follows: teneral of parental generation → immature of parental generation prior to aestivation → immature of parental generation after aestivation → mature of parental generation → spent of parental generation prior to hibernation (Fig. 1B). In other ‘autumn breeders’ (Type 3 according to Thiele 1977), the same order of physiological conditions of the adults is observed, but without an aestivation parapause. As in the previous case, the abundance of species can vary widely. For example, in the grass-forb steppe, the abundance of *Calathus ambiguus* was about 500 individuals in June and August (Fig. 3A), but in the sagebrush-grassland desert steppe, the abundance of *Pseudotaphoxenus rufitarsis major* was only 41 and 36 individuals at the end of September – beginning of October, respectively (Fig. 3B), yet the sex and age structure in the populations of both species was complete.

Importantly, in all these cases there are clear changes in successive waves of activity of different adult ‘age’ groups. It should be noted that in populations of many carabid species, the individuals of ancestral generations (which live and breed during two or more years) are often represented. In these cases the pattern of change in the physiological conditions can be blurred because separate successive waves of activity overlap each other.

Thus, it is not abundance, but rather a regular change in the physiological condition that allows for a reconstruction of the life cycle at the local population scale, and this must be regarded as the criterion for the successful existence and breeding of a population in a particular habitat. Species that meet these demands are considered ‘residents’ and their habitats ‘residential’.

**Figure 4.**
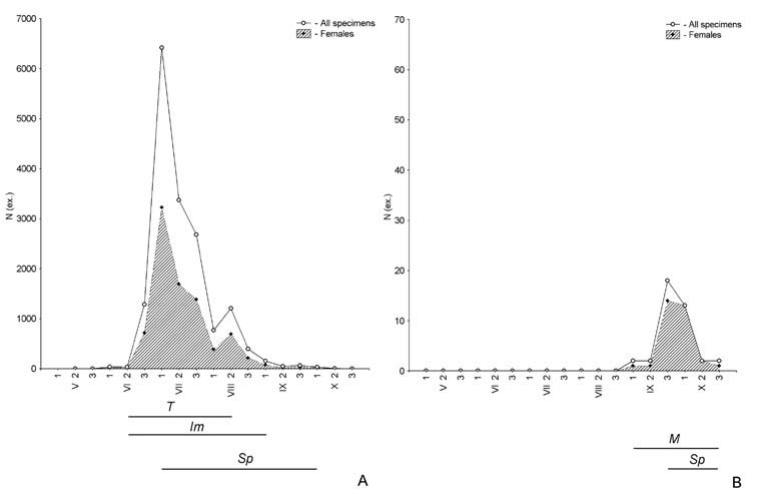
Seasonal dynamics of activity, as well as the age structure of the populations of *Harpalus rufipes* from reedbeds along the River Khara (**A**) and *Pseudotaphoxenus rufitarsis major* from the lakeside salt-marsh (**B**), in 2006 (see Figure 2 for further explanations).

An incomplete demographic spectrum of a population means that the probability of a complete life cycle in a particular habitat is low to zero. Such a situation is often followed by extremely high abundance levels. In reedbeds from the end of June until the end of July, *Harpalus rufipes* was by far the most numerous carabid beetle collected, with abundance levels of 1753, 7047, 3770 and 2830 for successive ten-day periods. Without information on the physiological conditions of individuals, this species may be considered dominant in this habitat. However, mature females were completely absent from the demographic spectra in this local population of *Harpalus rufipes*. Moreover, there were no successive waves of activity, because the peaks of abundance in teneral, immature and spent beetles were observed at the same time (Fig 4A). In these cases a reproductive phase in the demographic spectra of the local populations was absent.

Yet the presence of mature specimens is not necessarily evidence of successful breeding. For example, in lakeside salt-marshes, the demographic spectrum of *Pseudotaphoxenus rufitarsis major* was mainly represented by mature specimens. The abundance of spent beetles was very low, while teneral and immature beetles were completely absent (Fig. 4B). The lack of young specimens in the demographic spectrum of this species provides evidence of immigration of mature beetles. Species with incomplete demographic spectra are here considered ‘migrants’ and their habitats as ‘transit’.

The spatial distribution of carabid species is determined by the availability both of habitats and landscape suitable for the complete realization of their life cycle. So the same habitat can be residential for one species and transit for another. Among the examples discussed above, reedbed is a residential habitat for *Pseudotaphoxenus transfuga* (Fig. 2A), but a transit habitat for *Harpalus rufipes* (Fig. 4A). At the same time, various habitats offer different living conditions to the same species. The sagebrush-grassland desert steppe on the northern slope of the Ulagan Mountain is a residential habitat for *Pseudotaphoxenus rufitarsis major* (Fig. 3B), while the lakeside salt-marsh is a transit one for this species (Fig. 4B).

In summary, the demographic structures of 66 carabid species found in the Lake Elton region were analyzed. The other 109 carabid species were represented by only one or two individuals (Appendix). Considering the differences in abundance and demographic structure of the populations, three groups of Carabidae of the studied habitats can be distinguished:

Residents with their life cycles completed in a given habitat. In such species, migration forms only a facultative part of the life cycle. The catches of different species vary widely and sometimes differ by two orders of magnitude.

Migrants that are characterised by relatively high numbers, yet rarely dominant, but with an incomplete demographic structure in particular habitats. Because their reproduction and development are observed in different habitats, their roles in specific assemblages would be minor. Migration forms both facultative and obligatory parts of their life cycles.

Sporadic species with very low numbers, probably not associated with a particular habitat, neither during migration nor reproduction.

**Figure 5.**
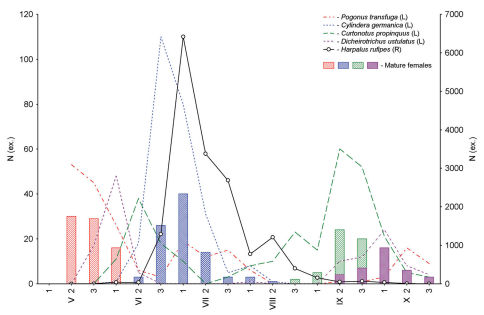
Seasonal variation in abundance curves and reproduction aspects in four resident carabid species coupled with abundance of a migrant-species *Harpalus rufipes* from reedbeds, combined data for 2006/07 (**R** and **L** right and left Y axis, respectively; **N (ex.)** – number of specimens).

Without question, residents interact both with their prey and with each other in a particular habitat. Sporadic species are hardly important to a carabid community because of their low abundance levels. The role of migrants in the local carabid community remains unknown, with possible interactions between the migrants and residents. First, even very high numbers of migrants in relatively small-sized habitats do not reflect the condition of the populations of other carabid species. For example, in reedbeds of an area of 1 km2, more than 13 000 specimens of *Harpalus rufipes* were trapped. This equates to a population density of about six individuals per square meter. This is a very high value. For example, the pest threshold of *Zabrus tenebrioides*, which is of the same size as *Harpalus rufipes*, is two-three individuals per square meter. Hence, if the captured specimens of *Harpalus rufipes* fed in this habitat and interacted with other species, we would expect changes in the demographic parameters of residents during this period. However, this is not the case, because the dynamics of the demographic structure in the populations of resident carabid beetles failed to change during this period (Fig. 5). Second, relatively high numbers and species diversity levels of migrants were recorded at some seemingly unsuitable sites. These sites included the lakeside salt-marsh with high salt concentrations, poor vegetation and soil, as well as occasional floods. Under these conditions, only some specialist Carabidae: 17 species from the genera *Cephalotha,Calomera,Tachys,Bembidion,Pogonus,Pogonistes,Cardiaderus,Dyschiriodes,Poecilus,Daptus,Dicheirotrichus* and *Harpalus*, can survive. Among 66 species collected in this habitat, 75% can neither feed nor breed there (see Tables 1–2 and Appendix). Nonetheless, the catching efficiency of several migrants (for example the bothrobiont *Pseudotaphoxenus rufitarsis major*) in this habitat was not lower compared to that in zonal sites.

“Stable” and “labile” components can be recognized in ground-beetles communities (Makarov and Matalin 2009). The former includes species whose life cycles are realized in certain habitats (residents), while the latter comprises species that are not capable of breeding in particular habitats (migrants and sporadic species).

**Figure 6.**
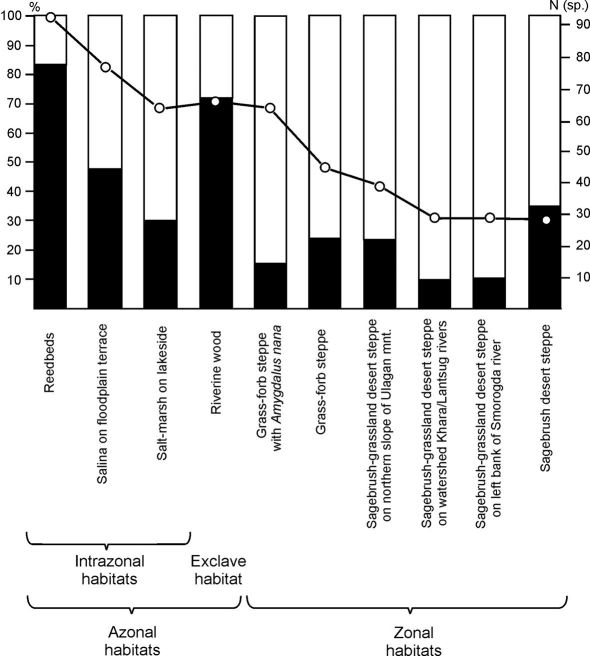
Species diversity and the share of labile/stable components in particular habitats in the Lake Elton region, combined data for 2006/07 (black bars – labile component, white bars – stable component, line – number of species; **N (sp.)** – number of species).

The ratio of stable to labile components in the studied habitats varied strongly and was not always in favour of residents. Resident species comprised only 6–35% of the species list and 15–90% of total abundance. In zonal habitats, residents formed the dominant part of the assemblage. More than 65% of total abundance and 15–35% of total species diversity consisted of resident species. In azonal habitats the labile component prevailed. These species accounted for about 75% of the fauna and about 80% of total abundance (Fig. 6). Only in zonal habitats did results from pitfall trapping adequately reflect the state of the carabid community while azonal and apparently disturbed habitats are only transit sites for many species of ground beetles.

## Discussion

According to our data, the capture in a pitfall trap indicates only the fact that the beetle has moved across the trap area, but do not reflect true abundances. In some cases, errors occurring from direct interpretations of pitfall trapping data can be severe, and statistical techniques can not compensate for this. This is evident from cases in which high numbers of some carabid species are collected from seemingly unsuitable locations, for example from city dumps (Budilov 2002; Romankina et al. 2007), urban quarters (Khotuleva 1997; Sharova and Kiselev 1999), places with strong oil or chemical pollution (Avtaeva 2006) and along roads (Noordijk et al. 2008; Solodovnikov 2008). The varying contribution of the labile component substantially distorts our knowledge of species diversity in carabid communities. Taking into account the contribution of the labile component can change conclusions based on pitfall trapping data considerably.

**Figure 7.**
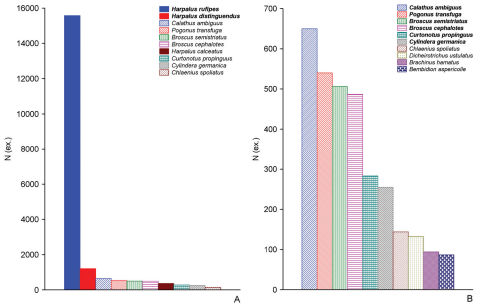
Numbers of the 10 most abundantly collected carabid species in reedbeds with regards to migrants (**A**) and residents only (**B**). Dominant species are in bold text, combined data for 2006/07; **N (ex.)** – number of specimens (after Makarov and Matalin 2009).

Firstly, criteria for determining the most abundant, or dominant species inevitably vary. The abundance of migrants in some cases is one order of magnitude higher than that of residents. Therefore, estimating the faunistic or community features based solely on abundant or dominant species, fail to solve the problem and can even worsen the situation. In reedbeds, for example, 36 migrant species made up about 83% of the total abundance. The complex of dominants in this community, as identified by the usual criterion (abundance exceeding 5%) while discarding the demography of individual species, contains only two polyzonal migrants *Harpalus rufipes* and *Harpalusdistinguendus*. In fact, six thermophilic resident species form the main body of this community: *Calathus ambiguus*, *Pogonus transfuga*, *Broscus semistriatus*, *Broscus cephalotes*, *Curtonotus propinguus* and *Cylindera germanica* (Fig. 7).

Secondly, common information regarding the habitat preferences of particular species, as well as indicator species, is considerably altered. In our case, all studied habitats belong to two contrasting groups: dry desert steppes and riparian, more or less halophilic habitats. As such, variation in carabid populations is expected. When analyzing the habitat distribution of all dominants-subdominants, we find more or less eurytopic species inhabiting both zonal dry steppes on floodplain terraces and azonal alluvial salt-marshes. The grouping of dry steppes is very poor and contains one or two species which occur in one to three habitats, as a rule. In contrast, the inhabitants of salt-marshes are very diverse and peculiar. Interestingly, the woodland in the ‘Biological’ Ravine supports not only a native carabid beetle community, but also a peculiar species, *Harpalus zabroides* (Table 1). Results from an analysis of the habitat distribution based solely on residents are distinctly different. Only one species, *Calathus ambiguus*, can be labelled eurytopic because it reproduces in nine of the ten studied habitats. The communities of carabid beetles on floodplain terraces and in flood-plains are clearly isolated from each other. Each of them includes the main body of oligotopic species and a few stenotopic ones. Finally, the riverine woodland does not have a native carabid community and can be considered a transit habitat for practically all carabid species (Table 2).

As such, the contribution of migrants to the trophic structure of carabid communities is not apparent and requires further research. That a particular carabid species inhabits and breeds in, and even dominates a certain habitat, is only a hypothesis that needs corroboration each time. Species with high abundance levels and high frequency of occurrence in a particular habitat can belong to both labile and stable components. Thus, in the Lake Elton region, *Calosoma auropunctatum*, *Dolichus halensis*, *Amara aenea*, *Harpalus calceatus*, *Harpalus rufipes*, *Harpalus distinguendus* and *Anisodactylus signatus* belong to the labile component in all the habitats where they occur; *Cephalota elegans* comprises the main element of the stable component in several azonal habitats; while *Calathus ambiguus*, *Cymindis lateralis* and *Pseudotaphoxenus rufitarsis maior* play the main role in the composition of the stable component in the majority of zonal habitats (Appendix). Overall, 65–75% of the species diversity of both individual habitats and the landscape as a whole comprised of non-residential species. It is important to note that almost half of the migrants (41 of 94 species) failed to breed in any of the studied habitats. Thus, the distances of their movements are substantially greater compared to the size of the site. So, the migrations of such species should be characterised at the landscape scale.

**Table 1.** Habitat preferences of individual species and the composition of carabid assemblages with the labile component dominant and subdominant species (combined data for 2006/07).

*Species*	*Zonal habitats*						*Azonal habitats*			
							*Exclave habitat*	*Intrazonal habitats*		
	*Left bank of River Bol’shaya Smorogda*		*Northern slope of Ulagan Mnt.*	*Right bank of River Khara*			*Riverine wood in “Biological” Ravine*	*Reedbeds along Rever Khara*	*Salina on floodplain terrace of River Khara*	*Lakeside salt-marsh*
	*Sagebrush desert steppe*	*Sagebrush-grassland desert steppe*	*Sagebrush-grassland desert steppe*	*Sagebrush-grassland desert steppe*	*Grass-forb steppe*	*Grass-forb steppe with Amygdalus nana*				
*Calathus ambiguus*	1									
*Pseudotaphoxenus rufitarsis major*	1									1
*Harpalus rufipes*	1					1				
*Harpalus distinguendus*	1			1						
*Cymindis lateralis*	1			1		1	1			
*Curtonotus desertus*	1		1							
*Amara ambulans*	1					1	1			
*Carabus bessarabicus concretus*	1					1				
*Cephalota atrata*	1									
*Cicindela campestris*				1						
*Harpalus anxius, H. picipennis*					2					
*Amara aenea*					1					
*Poecilus punctulatus*						1				
*Calathus distinguendus*, *Cymindis lineata*						2	2			
*Harpalus zabroides*							1			
*Amara ingenua*						1		1		
*Broscus semistriatus*, *Harpalus calceatus*							2	2		
*Dicheirotricus ustulatus*								1		
*Pogonus transfuga*, *Chlaenius spoliatus*								2		2
*Cylindera germanica*, *Calosoma auropunctatum*, *Dyschiriodes luticola*, *Dolichus halensis*, *Curtonotus propinquus*, *Brachinus hamatus*								6		
*Cephalota elegans*									1	
*Clivina ypsilon*, *Broscus cephalotes*, *Bembidion aspericolle*, *Tachys scutellaris*, *Poecilus cupreus*, *Pterostichus niger*, *Amara littorea*, *Amara similata*, *Anisodactylus poeciloides*, *Anisodactylus signatus*								10		
*Cephalota chiloleuca*, *Scarites tericola*, *Bembidion minimum*, *Pogonus meridionalis*, *Agonum gracilipes*, *Harpalus smaragdinus*, *Cymindis decora*									7	
*Pogonus cumanus*, *Pogonistes convexicollis*, *Pogonistes rufoaeneus*, *Cardiaderus chloroticus*, *Daptus vittatus*										5

## Conclusions

Because we have only very few examples that illustrate more or less close relations between ground beetles and their habitats, we are unable to assess the commonality of the situation described in the present study. However, it is conceivable that migrants in a carabid beetle community contribute to diversity estimates. Based on results from this study, some preliminary conclusions can be made.

A study of the demographic structure of local populations and an assessment of the migratory/residential status of particular carabid species are possible ways to increase the reliability of pitfall trapping information.

Up to 65–75% of species diversity, both of particular habitats and the landscape as a whole, can comprise of non-residential carabid species, i.e. migrants.

Results from pitfall traps adequately reflect the state of carabid communities only in zonal habitats. Azonal and apparently disturbed habitats are only transit sites for many species of ground beetles.

Knowledge concerning the composition of carabid communities, as well as study techniques, need to be significantly updated. No statistical method is capable of correcting the errors inferred from direct interpretations of pitfall trapping results.

**Table 2.** Habitat preferences of individual species and the composition of carabid assemblages without the labile component resident species only (combined data for 2006/07).

*Species*	*Zonal habitats*						*Azonal habitats*			
							*Exclave habitat*	*Intrazonal habitats*		
	*Left bank of River Bol’shaya Smorogda*		*Northern slope of Ulagan Mnt.*	*Right bank of River Khara*			*Riverine wood in “Biological” Ravine*	*Reedbeds along Rever Khara*	*Salina on floodplain terrace of River Khara*	*Lakeside salt-marsh*
	*Sagebrush desert steppe*	*Sagebrush-grassland desert steppe*	*Sagebrush-grassland desert steppe*	*Sagebrush-grassland desert steppe*	*Grass-forb steppe*	*Grass-forb steppe with Amygdalus nana*				
*Calathus ambiguus*	1									
*Cicindela campestris*, *Carabus bessarabicus concretus*, *Pseudotaphoxenus rufitarsis major*, *Curtonotus desertus*	4									
*Taphoxenus gigas*	1		1							
*Cymindis lateralis*	1					1				
*Poecilus sericeus*	1			1						
*Cephalota atrata*	1			1						
*Ophonus minimus*	1			1						
*Harpalus cyclogonus*, *Harpalus anxius*		2			2					
*Harpalus serripes*			1							
*Amara ambulans*, *Amara diaphana*, *Brachinus costatulus*			3							
*Harpalus foveiger*				1						
*Harpalus picipennis*, *Harpalus zabroides*					2					
*Poecilus punctulatus*, *Harpalus calathoides*					2					
*Cymindis lineata*						1				
*Calathus distinguendus*						1	1			
*Pogonus transfuga*, *Pogonistes rufoaeneus*, *Tachys scutellaris*, *Broscus semistriatus*, *Dyschiriodes salinus striatopunctatus*								5		
*Cylindera germanica*, *Scarites terricola*, *Dischiriodes luticola*, *Poecilus nitens*, *Curtonotus propinguus*, *Dicheirotrichus ustulatus*, *Brachinus hamatus*								7		
*Cephalota elegans*, *Pogonus meridionalis*, *Pogonus punctulatus*, *Daptus vittatus*, *Harpalus dispar splendens*									5	
*Chlaenius spoliatus*								1		1
*Carabus clathratus*, *Clivina ypsilon*, *Broscus cephalotes*, *Bembidion aspericolle*, *Anisodactylus poeciloides*, *Acupalpus elegans*, *Acupalpus parvulus*, *Stenolophus mixtus*, *Dicheirotrichus discicollis*, *Chlaenius tristis*								10		
*Cephalota chiloleuca*, *Dyschiriodes cylindricus hauseri*, *Amara abdominalis*, *Amara parvicollis*									4	
*Calomera littoralis conjuctaepustulata*, *Dyschirius humeratus*, *Pogonus cumanus*, *Pogonistes convexicollis*, *Pogonistes angustus*, *Cardiaderus chloroticus*										6

## Appendix

**Table:** Classification of the carabid species from the Elton Lake region in migrant (M), resident (R) and sporadic (S) species, based on their abundance and demographic spectrum in each habitat type (combined data for 2006/07).

*HABITATSSPECIES*	*Zonal habitats*						*Azonal habitats*			
							*Exclave habitat*	*Intrazonal habitats*		
	*Left bank of River Bol’shaya Smorogda*		*Northern slope of Ulagan Mountain*	*Floodplain terrace of River Khara*		*Watershed of the Rivers Khara and Lantsug*	*“Biological” Ravine*	*Bank of River Khara*	*Floodplain terrace of River Khara*	*Lakeside near the mouth of River Khara*
	*Sagebrush desert steppe*	*Sagebrush-grassland desert steppe*	*Sagebrush-grassland desert steppe*	*Grass-forb steppe with Amygdalus nana*	*Grass-forb steppe*	*Sagebrush-grassland desert steppe*	*Riverine wood*	*Reedbeds*	*Salina*	*Salt-marsh*
*Cylindera (Cylindera) germanicagermanica* (Linnaeus, 1758)	m			m				R	R	M
*Cylindera (Eugrapha) contorta contorta* (Fischer von Waldheim, 1828)										s
*Cephalota (Taenidia) elegans elegans* (Fischer von Waldheim, 1823)								m	R	R
*Cephalota (Taenidia) atrata* (Pallas, 1776)	R	R		m	m	R			m	m
*Cephalota (Taenidia) chiloleuca* (Fischer von Waldheim, 1820)									R	
*Calomeralittoralis conjunctaepustulata* (Dokhtouroff, 1887)										R
*Cicindela* (s. str.) *campestris pontica* Fischer von Waldheim, 1828	R	R	R	R	R	R		M	M	
*Cicindela* (s. str.) *maritima kirgisica* Mandl, 1936										s
*Notiophilus laticollis* Chaudoir, 1850				s	s					
*Calosoma (Campalita) auropunctatum dzungaricum* Gebler, 1833			m	m			M	M	M	M
*Calosoma (Caminara) denticolle* Gebler, 1833				m			M	M	M	m
*Calosoma (Charmosta) investigator investigator* (Illiger, 1798)							s		s	
*Carabus (Limnocarabus) clathratus clathratus* Linnaeus, 1761							m	R		
*Carabus (Tomocarabus) bessarabicus concretus* Fischer von Waldheim, 1823	R	R	R	R	R	R	M	M	M	
*Elaphrus* (s.str.) *hypocrita hypocrita* Semenov, 1926							s	s		
*Scarites (Parallelomorphus) terricola terricola* Bonelli, 1813								R	R	M
*Clivinaypsilon* Dejean, 1829								R	m	
*Dyschirius* (s. str.) *humeratus* Chaudoir 1850									M	r
*Dyschiriodes (Eudischirius) globosus* (Herbst, 1783)				s						
*Dyschiriodes (Eudischirius) rufipes* (Dejean, 1825)							s			
*Dyschiriodes (Chyridysus) euxinus* (Znojko, 1927)									s	
*Dyschiriodes (Chyridysus) rufimanus* (Fleischer, 1898)								s	s	
*Dyschiriodes* (s.str.) *apicalis* (Putzeys, 1846)								s		
*Dyschiriodes* (s.str.) *chalceus* (Erichson, 1937)								S	S	
*Dyschiriodes* (s.str.) *cylindricus hauseri* (Fleischer, 1898)								M	r	R
*Dyschiriodes* (s.str.) *luticola luticola* (Chaudoir, 1850)								R	R	M
*Dyschiriodes* (s.str.) *pusillus* (Dejean, 1825)										s
*Dyschiriodes* (s.str.) *salinus striatopunctatus* (Putzeys, 1846)							m	r	r	r
*Dyschiriodes* (s.str.) *tristis* (Stephens, 1828)						s		s		
*Broscus cephalotes* (Linnaeus, 1758)							M	R	M	m
*Broscus semistriatus* (Dejean, 1828)	M		M	M	M		M	R	R	R
*Trechus* (s. str.) *quadristriatus* (Schrank, 1781)							s			s
*Tachys* (s.str.) *scutellaris* (Stephens, 1829)								R	R	R
*Paratachys bistriatus* (Duftschmid, 1812)									R	R
*Paratachyscentriustatus* Reitter, 1894								s		s
*Paratachysmicros* (Fischer von Waldheim, 1828)								S		S
*Bembidion (Notaphus) varium* (Olivier, 1795)								s	s	
*Bembidion (Philochtus) pallidiveste* Carret, 1905	m			m				m		m
*Bembidion (Emphanes) minimum* (Fabricius, 1792)									R	
*Bembidion (Talanes) aspericolle* (Germar, 1829)								R	m	
*Bembidion (Trepanes) octomaculatum* (Goeze, 1777)							s		s	
*Bembidion* (s. str.) *qudrimaculatum* (Linnaeus, 1761)								s		
*Cardiaderus chloroticus* (Fischer von Waldheim, 1823)										R
*Pogonus (Pogonoidius) cumanus* Lutshnik, 1916									m	R
*Pogonus (Pogonoidius) meridionalis* Dejean, 1828									R	R
*Pogonus (Pogonoidius) punctulatus* Dejean, 1828									R	R
*Pogonus* (s. str.) *iridipennis* Nicolai, 1822									s	s
*Pogonus* (s. str.) *luridipennis* (Germar, 1823)									s	s
*Pogonus* (s. str.) *orientalis* Dejean, 1828									s	
*Pogonus* (s. str.) *transfuga* Chaudoir, 1871	m				m			R	R	R
*Pogonistes* (s. str.) *angustus* (Gebler, 1829)										R
*Pogonistes* (s. str.) *convexicollis* Chaudoir, 1871									M	R
*Pogonistes* (s. str.) *rufoaeneus* (Dejean, 1828)								R	R	R
*Poecilus* (s. str.) *cupreus cupreus* (Linnaeus, 1758)				M		m	m	M	M	m
*Poecilus* (s. str.) *punctulatus* (Schaller, 1783)				R	R			M	M	
*Poecilus* (s. str.) *sericeus sericeus* (Fischer von Waldheim, 1823)	R	M		R	R	R		M		
*Poecilus (Ancholeus) laevicollis* Chaudoir, 1842				s				s		
*Poecilus (Ancholeus) nitens nitens* Chaudoir, 1850	M	M						r	r	M
*Poecilus (Ancholeus) puncticollis* (Dejean, 1828)								M		M
*Pterostichus (Platysma) niger niger* (Schaller, 1783)							M	M		
*Pterostichus (Argutor) leonisi* Apfelbek, 1904										s
*Pterostichus (Adelosia) macer macer* (Marsham, 1802)			M	M			M	M		
*Pterostichus (Pseudomaseus) piceolus* (Chaudoir, 1850)				s						
*Pterostichus (Phonias) taksonyis* Csiki, 1930										s
*Pterostichus (Morphnosoma) melanarius melanarius* (Illiger, 1798)							s	s		
*Calathus* (s. str.) *distinguendus* Chaudoir, 1846				R			R	M		
*Calathus (Neocalathus) ambiguusambiguus* (Paykull, 1790)	R	R	R	R	R	R	R	R	R	M
*Calathus (Neocalathus) melanocephalus melanocephalus* (Linnaeus, 1758)				m			M	M	M	
*Dolichus halensis* (Schaller, 1783)						M	m	M	M	M
*Pseudotaphoxenusrufitarsis major* Tschitscherine, 1895	R	R	R	R	R	R	M	M	M	M
*Taphoxenus* (s. str.) *gigas* (Fischer von Waldheim, 1823)	R	M	R	R	R	R	M	M		M
*Agonum* (s. str.) *gracilipes* (Duftschmid, 1812)				M			M	r	M	
*Agonum (Agonothorax) lugens* (Duftschmid, 1812)				s				s		
*Agonum (Agonothorax) sexpunctatum* (Linnaeus, 1758)								s		
*Agonum (Europhilus) thoreyi thoreyi* (Dejean, 1828)								s		
*Platynus* (s. str.) *longiventre* Mannerheim, 1825							s			
*Anchomenus* (s. str.) *dorsalis* (Pontoppidan, 1763)							s			
*Amara (Zezea) chaudoiri chaudoiri* Putzeys, 1858								s		
*Amara* (s. str.) *aenea* (De Geer, 1774)		M		M	M		M	M	M	M
*Amara* (s. str.) *eurynota* (Panzer, 1796)				M			M	M	M	
*Amara* (s. str.) *littorea* C.G. Thomson, 1857				M			M	r	M	M
*Amara* (s. str.) *littoralis* Mannerheim, 1843					s					
*Amara* (s. str.) *similata* (Gyllenhal, 1810)				M			M	M	M	M
*Amara* (s. str.) *tibialis* (Paykull, 1798)		M		M			M			
*Amara (Celia) bifrons* (Gyllenhal, 1810)				M			M	M	M	
*Amara (Celia) municipalis municipalis* Duftschmid, 1812							s			
*Amara (Celia) saginata* (Menétries, 1849)	M			M						
*Amara (Xenocelia) ingenua* (Duftschmid, 1812)			M	M	M	M	M	M	M	M
*Amara (Xenocelia) ambulans* C. Zimmermann, 1832	M	M	R	M	M	M	M	M	M	M
*Amara (Paracelia) saxicola* C. Zimmermann, 1832	M	M	M	M	M	M	M		M	M
*Amara (Bradytus) apricaria* (Paykull, 1790)							M	M	M	M
*Amara (Bradytus) consularis* (Duftschmid, 1812)		M					M			M
*Amara (Percosia) pastica* Dejean, 1831			s							
*Amara (Amathitis) abdominalis* (Motschulsky, 1844)								M	R	M
*Amara (Amathitis) parvicollis* Gebler, 1833									R	
*Amara (Ammoxena) diaphana* Tschitschérine, 1894			R	M						
*Curtonotus* (s. str.) *aulicus* (Panzer, 1796)							M	M		
*Curtonotus* (s. str.) *desertus* Krynicki, 1832	R	R	R	R	R	R	M			
*Curtonotus* (s. str.) *convexiusculus* (Marsham, 1802)								M		
*Curtonotus* (s. str.) *propinquus* (Menétries, 1832)							M	R	R	
*Curtonotus (Ammoleirus) megacephalus* (Gebler, 1829)			s							
*Anisodactylus (Pseudanisodactylus) signatus* (Panzer, 1796)				M	M		M	M	M	M
*Anisodactylus* (s. str.) *binotatus* (Fabricius, 1787)								M		M
*Anisodactylus (Hexatrichus) poeciloides pseudoaeneus* Dejean, 1829								R	M	M
*Diachromus germanus* (Linnaeus, 1758)								s		
*Bradycellus* (s. str.) *caucasicus* (Chaudoir, 1846)					s					
*Dicheirotrichus* (s. str.) *ustulatus* (Dejean, 1829)			M					R	R	M
*Dicheirotrichus (Trichocellus) discicollis* (Dejean, 1829)								R		
*Dicheirotrichus (Trichocellus) stenothorax* (Kabak & Kataev, 1993)								s		
*Stenolophus* (s. str.) *mixtus* (Herbst, 1784)							M	R		m
*Acupalpus* (s. str.) *elegans* (Dejean, 1829)								R		
*Acupalpus* (s. str.) *exiguus* Dejean, 1829	s									
*Acupalpus* (s. str.) *meridianus* (Linnaeus, 1761)			M				M			M
*Acupalpus* (s. str.) *parvulus* (Sturm, 1825)	M							R	M	M
*Daptusvittatus* Fischer von Waldheim, 1823			M					M	R	R
*Harpalus (Cephalophonus) cephalotes* Fairmaire & Laboulbene, 1854				M			M	m	m	m
*Harpalus (Pseudophonus) griseus* (Panzer, 1796)							M	M	M	
*Harpalus (Pseudophonus) rufipes* (De Geer, 1774)	M	M	M	M	M	M	M	M	M	M
*Harpalus (Pseudophonus) calceatus* (Duftschmid, 1812)	m		m	m	m		M	M	M	M
*Harpalus (Semiophonus) signaticornis* (Duftschmid, 1812)			M		M					
*Harpalus* (s. str.) *cyclogoniuscyclogonius* Chaudoir, 1844		R		R	R	M				
*Harpalus* (s. str.) *rubripes* (Duftschmid, 1812)			M	M	M		m	m	M	M
*Harpalus* (s. str.) *politus politus* Dejean, 1829					s					
*Harpalus* (s. str.) *serripes serripes* (Quensel, 1806)			R	M	R	R		M	M	
*Harpalus* (s. str.) *pumilus* Sturm, 1818						s				
*Harpalus* (s. str.) *picipennis* Duftschmid, 1812		M		M	R		M			
*Harpalus* (s. str.) *amplicollis* Menétries, 1848					M	M		M	M	M
*Harpalus* (s. str.) *anxius* (Duftschmid, 1812)		R		R	R	M				
*Harpalus* (s. str.) *calathoides* Motschulsky, 1844	M	M	M	R	R	M	M		M	
*Harpalus* (s. str.) *froelichi* Sturm, 1818				s						
*Harpalus* (s. str.) *hirtipes* (Panzer, 1796)			s							
*Harpalus* (s. str.) *zabroides* Dejean, 1829				M	R	M	M	M	M	M
*Harpalus* (s. str.) *tardus* (Panzer, 1796)				M						
*Harpalus* (s. str.) *oblitus oblitus* Dejean, 1829	M	M	M	M	M	M	M		M	
*Harpalus* (s. str.) *fuscipalpis* Sturm, 1818	M	M		M	M		M	M	M	
*Harpalus* (s. str.) *inexpectatus* Kataev, 1989				s						
*Harpalus* (s. str.) *smaragdinus* (Duftschmid, 1812)		M	M	M	M			m	m	M
*Harpalus* (s. str.) *autumnalis* (Duftschmid, 1812)			s			s				
*Harpalus* (s. str.) *foveiger* Tschitschérine, 1895	M	M			R	R				
*Harpalus* (s. str.) *dispar splendens* (Gebler, 1829)			M					M	R	R
*Harpalus* (s. str.) *circumpunctatus* Chaudoir, 1846			M	M				M		M
*Harpalus* (s. str.) *steveni* Dejean, 1829					s				s	
*Harpalus* (s. str.) *terrestris* (Motschulsky, 1844)			M	M						
*Harpalus* (s. str.) *distinguendus distinguendus* (Duftschmid, 1812)	M	M	M	M	M	M	M	M	M	M
*Microderes* (s. str.) *brachypus* (Steven, 1809)	M	M	M			M		M		
*Acinopus (Haplacinopus) striolatus* Zoubkoff, 1833			s			s				
*Ophonus (Metophonus) laticollis* Mannerheim, 1825							s			
*Ophonus (Hesperophonus) azureus* (Fabricius, 1775)				M	r		m	M		
*Ophonus (Hesperophonus) minimus* Motschulsky, 1845	R	M		M	R	R	M			
*Ophonus (Hesperophonus) convexicollis* Menétries, 1832					s	s				
*Dixus eremita* (Dejean, 1825)			s							
*Dinodes decipiens* (L. Dufour, 1820)		s		s						
*Chlaenius (Chlaenites) spoliatus spoliatus* (P. Rossi, 1792)				M			M	R	M	R
*Chlaenius (Chlaeniellus) nigricornis* (Fabricius, 1787)								s		
*Chlaenius (Chlaeniellus) terminalis* Dejean, 1826									s	
*Chlaenius (Chlaeniellus) tibialis* Dejean, 1826								s		
*Chlaenius (Chlaeniellus) tristis tristis* Schaller, 1783								R	M	M
*Oodes (Lachnocrepis) prolixus* (H. Bates, 1873)									s	s
*Syntomus pallipes* (Dejean, 1825)							s			
*Microlestes badulini* Komarov, 1989									s	
*Microlestes fissuralis* (Reitter, 1901)				s	s	s	s			
*Microlestes maurusmaurus* (Sturm, 1827)			s	s						
*Cymindis* (s. str.) *decora* Fischer von Waldheim, 1829							M	M	M	
*Cymindis* (s. str.) *picta picta* (Pallas, 1771)				M			M	M	M	
*Cymindis* (s. str.) *lineata* (Quensel, 1806)				r	M		M			M
*Cymindis* (s. str.) *scapularis scapularis* Schaum, 1857							s			
*Cymindis (Menas) miliaris* (Fabricius, 1801)				s	s		s	s		
*Cymindis (Tarsostinus) lateralis* Fischer von Waldheim, 1820	R	R	R	R	M	M	M	M	M	M
*Polystichusconnexus* (Fourcroy, 1785)			s	s	s					
*Zuphium* (s. str.) *olens olens* (P. Rossi, 1790)				s						
*Zuphium* (s. str.) *testaceum* Klug, 1832						s				
*Brachinus (Brachinidius) costatulus* Quensel, 1806		M	R	M			M	M	M	
*Brachinus (Cnecostolus) bipustulatus* Quensel, 1806				s						
*Brachinus (Cnecostolus) hamatus* Fischer von Waldheim, 1828					M		M	R	R	
*Mastaxthermarum thermarum* (Steven, 1806)							s	s		
*TOTAL SPECIES*	*28*	*29*	*39*	*69*	*46*	*33*	*69*	*97*	*85*	*73*
